# Comparison of Public Health Investments of Various Countries Amid a Need for Greater Transparency: A Narrative Review

**DOI:** 10.7759/cureus.29687

**Published:** 2022-09-28

**Authors:** Fatima Hasan, Asmita Rannaware, Sonali G Choudhari

**Affiliations:** 1 Community Medicine, School of Epidemiology and Public Health, Jawaharlal Nehru Medical College, Datta Meghe Institute of Medical Sciences, Wardha, IND

**Keywords:** programs, disease, interventions, public health, economic investments, public health investments

## Abstract

A robust health system demands investments in public health and healthcare as they aid in closing the health protection gap. They are primarily responsible for longer life expectancies, disease prevention, and protection. Loopholes in the public health system were formed due to a lack of transparency and have only worsened throughout COVID-19. Spending more on public health is associated with fewer deaths, fewer food-borne illnesses, better sanitation, food safety, clean air and water, increased immunizations to stave against infectious diseases, and a decline in low birth weight. A comprehensive literature and data search was conducted using web-based search engines like PubMed, National Center for Biotechnology Information (NCBI), Google Scholar, Science Direct, and the New England Journal of Medicine. The review study standpoints healthcare spending, out-of-pocket expenditures, and other monetary use in various low-to-high-income countries, and the results are graphically represented. Countries with a strong public health system provide all the necessary aid to protect their citizens. They have cost-effective, readily available resources with fewer out-of-pocket expenditures (OOPs), government schemes, and health insurance to help their people. During our research, it was found how little the Indian government spends on healthcare as a percentage of gross domestic product (GDP) as compared to 'thought-to-be' poor countries like Bhutan.

## Introduction and background

The constant state of disease-spreading, which jeopardizes a person's health, leading to increased mortality and morbidity, should serve as enough warning about how lacking we are when it comes to a robust health system. The public health system is essential in safeguarding us against all unknown future manifestations, like severe diseases or outbreaks. When the public health system (a system that provides preventative, supportive, and curative services) gets severed, outbreak-like situations will arise sooner or later. Be it the ignorance on our part or the lack of funding by the government, people as a whole are affected, and the repercussions are gruesome. Because public health spans all sectors connected to health, determining where and why it is lacking remains a mystery. When we talk about equal advantages of health-related resources and commodities for everyone, we talk about public health. Providing health for all is a significant undertaking that involves considerable work, motivation, leadership, unity, financial assistance, and skilled personnel.

Even though public health is enormous, there are still challenges in attaining greater transparency in the area requiring investments. Though healthcare is delivered and managed locally [1], it is still a part of public health. Public health investments are becoming a more likely predictor of health outcomes as they determine how many resources and services are available [2]. Thus, comparing different healthcare investments of different countries will help us determine which country tops the healthcare benefit chart, having fewer out-of-pocket expenditures (OOPs) and more government funds to improve health [3].

Developing a solid healthcare system in a third-world or economically impoverished country is challenging. This review will be helpful for countries like India, where it was proved during COVID-19 that India's public health has collapsed [4]. An incompetent system might suggest how much divulgence we need to show to update our knowledge, infrastructure, resources, machinery, techniques, and mindset so that people believe in the system before anything else. As a developing country, India faces numerous challenges, which mainly require investments in quality and safety, resource use, efficient use of infrastructure, patient experience of care, and monetary constraints. Other challenges include good health insurance schemes, pandemic preparedness, adequate knowledge, and exposure to schemes [5]. As a result, this research focuses on why public health and its various components must remain a top priority and how we can implement cost-effective methods, absolving myths and barriers surrounding public health and bringing 'health above all' to another level.

## Review

A comprehensive literature search was conducted on the topic ‘Comparison of Public Health Investments of Various Countries With a Need for Greater Transparency’ using databases like PubMed, Google Scholar, and Science Direct. Search terms included economic investments, public health, interventions, disease, and programs. Nineteen articles written in English were chosen based on the criteria that quantified the need for investments in public health, the need for interventions, and why health must remain a priority. Global data from the Organization for Economic Co-operation and Development (OECD) website was used to assess the health expenditures done by the different countries. Articles ranging from 2008 to 2021 were used, reflecting how gradually the problem of lack of public health investments surfaced.

We studied the cost-effectiveness of appropriate public health initiatives, as well as what the major bottlenecks are and how the government might address them. A comparison was made between how much money low- and high-income countries spend on their healthcare systems and how much mandatory health insurance each country provides to its healthcare system. Other graphs were created to demonstrate how little money India's government spends on protecting its citizens. The articles were studied to determine whether there should be an increase in public health investments and total investments, highlighting problems such as the need for public health investment, potential hurdles to investment, and comparisons between elite and third-world nations' investments.

Articles are from developed countries that justified the need for investments and can be used to serve as a model. Three pieces of evidence were discovered, each from a different country, indicating that investments provide positive results. In addition, an investment chart is also drawn to highlight the subject. We found some articles with significant evidence. The data supporting public health spending was not only the cheapest, but it also produced the best results. In Australia, the National Preventive Health Taskforce published an evidence-based study that indicated that successful prevention has significant societal advantages, including higher economic productivity and performance [6-8]. In the United States, the Prevention for a Healthier America report says that investing in a community-based illness prevention program could yield a higher return on investment in five years. In the following decade or two, it could be much higher [8]. In Canada, healthy people, healthy performance, and healthy profits reported that shifting our focus to strategic investments in socioeconomic determinants of health will aid in improving future outcomes, with significant cost savings and other economic benefits [8]. In India, Kerala had a per capita expenditure of the public health department of Rupees 7,636, and it was the highest in the whole country [9].

**Figure 1:**
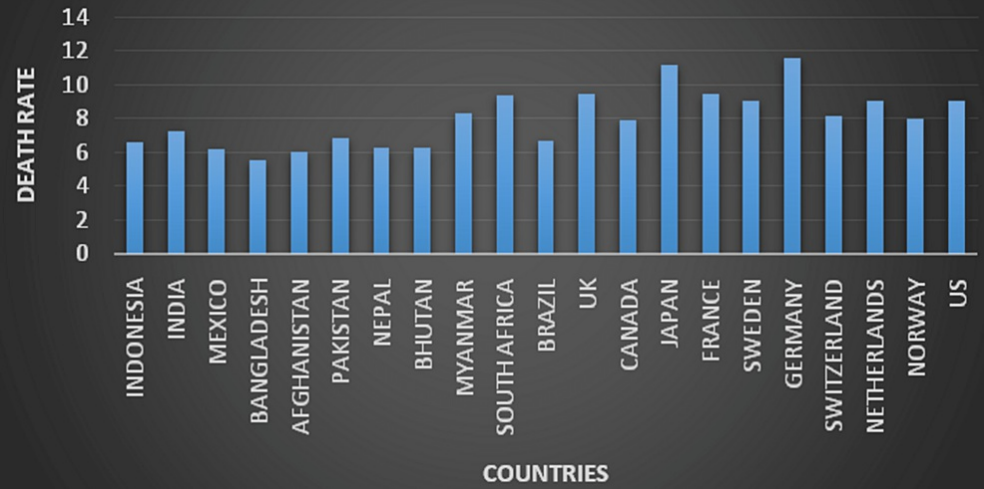
Death rate (per 1000 people) Data source [13]

**Figure 2:**
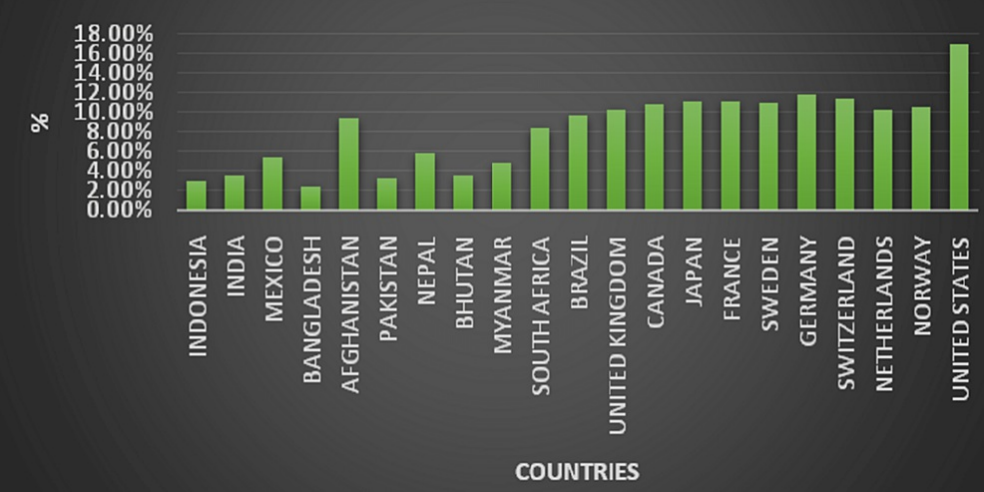
Health expenditure as a share of gross domestic product (GDP) in % Data source [12]

Table 1 provides information about the various countries' health expenditures, which include investments in government systems and health insurance [10]. Table 2 depicts the healthcare statistics of India like income level, money spent on health by the government, OOPs, health expenditure as a share of GDP, life expectancy, death rate, and the population of the country [11-14]. Figure 1 depicts the death rate of various countries per 1000 people. Despite how much governments invest, the death rate is uneven. The death rates of countries hiked significantly due to the Covid-19 pandemic [13]. Figure 2 shows health expenditure as a share of GDP. 'Thought-to-be' poor countries like Bhutan and Myanmar also have the same statistics as India [12]. Table 3 shows how income countries are defined by the World Bank and the various healthcare investments they have [15]. 

**Table 1:** A compendium of healthcare expenditure by various countries Data source [10]

Countries	Health expenditure from public funding in %, 2019		Health expenditure from public sources as a share of total government expenditure, 2019
	Government schemes/transfers	Health insurance	
Norway	86	-	18%
Sweden	85	-	19%
Japan	34	50	24%
United Kingdom	79	-	20%
New Zealand	70	9	18%
Germany	13	65	20%
France	6	71	15%
Italy	74	-	13%
Australia	69	-	16%
Russia	40	21	10%
China	29	28	8%
United States	39	12	22%
Mexico	27	23	10%
India	23	4	4%

**Table 2:** A summary of healthcare statistics Adapted from [11-14]

Name of the Country		Income level [11]	Money spent on health by the Government [12]	Out-of-pocket expenditure/voluntary (2018/2019) [12]	Health expenditure as a share of GDP in 2019 and 2020 [12]	Life expectancy (years of life at birth) [12]	Death rate (deaths per 1000 people) [13] 2021	Population, 2022 [14]
Indonesia		Lower-middle	$168 per capita	$168	2.9%	71.72 years	6.63	273,523,615
India		Lower-middle	$69.9 per capita	$186	3.6%	69.66 years	7.234	1,380,004,385
Mexico		Upper-middle	$558 per capita	$574	5.4%	75.1 years	6.21	128,932,753
Bangladesh		Lower-middle	$41.91 per capita	$81(2018)	2.34%(2018)	73.57 years	5.52	164,689,383
Afghanistan		Low	$49.84 per capita	$146.11(2018)	9.4%(2018)	65.98 years	6.04	38,928,346
Pakistan		Lower-middle	$42.87 per capita	$100.25(2018)	3.2% (2018)	67.79 years	6.80	220,892,340
Nepal		Low	$57.85 per capita	$91.64(2018)	5.84%(2018)	71.74 years	6.26	29,972,147
Bhutan		Lower-middle	$102.74 per capita	42.40(2018	3.6%(2018)	72.77 years	6.28	771,608
Myanmar		Lower-middle	$59.21 per capita	$223.02(2018)	4.79%(2018)	67.78 years	8.31	54,409,800
South Africa	Upper-middle		$475.6 per capita	$627	8.3%	64.13 years	9.36	59,308,690
Brazil	Upper-middle		$618.8 per capita	$848	9.6%	75.88 years	6.70	212,559,417
United Kingdom	High		$3533 per capita	$966	10.2%	81.4 years	9.45	67,886,011
Canada	High		$3,768 per capita	$1602	10.8%	82.1 years	7.88	36,708,083
Japan	High		$3,936 per capita	$754	11%	84.4 years	11.16	126,476,461
France	High		$4,414 per capita	$859	11.1%	82.9 years	9.46	65,273,511
Sweden	High		$4,712 per capita	$839	10.9%	83.2 years	9.08	10,099,265
Germany	High		$5,514 per capita	$1003	11.7%	81.4 years	11.57	83,783,942
Switzerland	High		$4,765 per capita	$2372	11.3%	84 years	8.12	8,654,622
Netherlands	High		4,742 per capita	$996	10.2%	82.2 years	9.04	17,134,872
Norway	High		$5,788 per capita	$956	10.5%	83 years	7.979	5,421,241
United States	High		$9,053 per capita	$1894	16.8%	78.9 years	9.04	331,002,651

**Table 3:** Income countries as defined by the World Bank Adapted from the Global Expenditure on Health by World Health Organization [15]

COUNTRIES	GOVERNMENT TRANSFERS	SOCIAL HEALTH INSURANCE	EXTERNAL AID	VOLUNTARY HEALTH INSURANCE	OUT-OF-POCKET SPENDING	OTHERS
LOW-INCOME	21%	1%	29%	2%	44%	3%
LOWER-MIDDLE	34%	7%	12%	3%	40%	3%
UPPER MIDDLE	38%	16%	1%	9%	34%	2%
HIGH-INCOME	48%	22%	-	5%	21%	4%

Discussion

Evolving Public Health

Public health operates under three principles: First, modifiable predictors of health, such as food, water, and physical and social environmental risks and threats that directly influence our health must be resolved and safeguarded. Second, no one should be denied life-saving interventions. When it comes to health, social class, status, economic background, race, and caste should never be considered. It should be illegal to deny someone the right to their well-being. Finally, disease prevention and optimal health should always be the top concern [16]. A crisis happens when there occurs a lack of capacity building and technical assistance. Whether simple or complex, humanitarian issues require interventions where providers combat both the growing disease and those already present [17]. A downtrend in infectious diseases, focus on prevention, reduced neonatal mortality rates, tackling antimicrobial resistance, improved nutrition, using digital health and artiﬁcial intelligence for social impact, and stronger government accountability are the things we need to resolve gradually.

The elite countries of the world focus on infection control, good infrastructure and workforce, improving reproductive health, beneficial health schemes, good quality of care for all, reducing poverty, using a team approach, and other technological advancements over third world countries to show a downward tendency in infectious diseases, decreasing newborn mortality rates, combating antimicrobial resistance, improving nutrition, leveraging digital health and artificial intelligence for social impact, and strengthening government accountability [18], So, taking them as an example we must enhance our efforts to gain health stability.

Focusing on Cost-Effectiveness and Out-of-Pocket Expenditures (OOPs)

Out of the total health budget, 15% is spent on primary healthcare. Under the pretext of health promotion and illness prevention, 85 per cent of the money goes to building major hospitals and purchasing utilities. To increase the cost-effectiveness, start by comparing overall health achievements to actual health spending in other nations. This might tell us how much they invested and what they gained out of that investment. A second technique is to look at each country's health expenditures and see if they are too high or too low compared to other countries' figures. This might tell us whether our investment is sufficient keeping in mind that the population level, income level, and statistics of every country vary. The third method would be to see if the current level and composition of health spending might be changed to produce a higher return in terms of outcome measures, such as disability-adjusted life years (DALYs) or increased consumption of non-health products, while keeping everything else the same [18].

When there isn't enough government investment, insurance schemes, or other goals to prevent additional concerns about a person's health, out-of-pocket payments rise. To reduce out-of-pocket expenditures, we must evaluate the importance of stewardship (which involves monitoring and regulating all healthcare-related demands), resource creation, health funding structures, and health care delivery. A sound healthcare delivery system helps provide equitable distribution of resources, available facilities and workforce to each citizen. These four criteria have been shown to aid in reducing out-of-pocket expenditures and can be employed explicitly, given the needs and our system's operation. Still, they will also assist us in ways to avoid wasteful expenditure and focus more on healthy spending [18].

The primary goal of this study was to determine public health policy and investments, which are often neglected. Those with the authority to make and carry out choices are unaware of its significance. Machines that are too old are overworked. Infrastructure is inadequate and ineffective. There are insufficient public health workers in villages and rural areas [16]. As one of the emerging countries, India faces numerous obstacles. We have a responsibility to safeguard people from infectious diseases that are always present, non-communicable conditions and their expanding risk factors that engulf individuals, and the ever-increasing lifestyle demand, as well as outbreaks/pandemics caused by pathogens that infect us [19]

Communicable diseases are the greatest threat of all time. Endemic diseases like HIV infection, AIDS, Tuberculosis (TB), malaria, and other tropical diseases continue to pose a national threat pre, peri and post coronavirus. All we know is that coronavirus taught us several lessons, the most important of which is to know your opponent [20]. Diseases, for example, necessitate preparation and a quick response time and identification. Diseases spread by vectors, such as dengue fever or acute encephalitis syndrome, must be targeted and well-planned. Taking command to safeguard oneself and one's surroundings comes before anything else; even if the government is taking many preventative precautions and conducting several initiatives and programs, taking control to protect oneself and one's surroundings must remain the highest priority [19].

The newly trending non-communicable diseases (NCDs), are also causing significant increases in mortality rates. NCDs not only make people vulnerable, but they also have an impact on the country's socioeconomic development. Each year, NCDs kill 41 million individuals worldwide, accounting for 71% of all deaths. Fourteen million people perish far too soon. The primary issue is that if we do not act with certainty and honesty, the number will rise to 55 million by 2030, which is only eight years away [21]. The statistic is disturbing and requires everyone's help to prevent it. Cardiovascular illnesses, malignancies, respiratory diseases, and diabetes are the most common NCDs [19].In India, cardiovascular disease and stroke are the leading causes of death for both men and women [22].

Other important diseases to be concerned about include maternal and newborn mortality, which are incredibly high. They should never be left in the dark and must remain a top priority. Thus, to prevent all these, we need a fluent, transparent, feasible and not misleading structure. Comparing investments done by various countries on health (Table 1) and collecting data on different types of investments by low- to high-income countries (Table 3) will give us some ideas about where we have to spend more and where we lack. This issue could only be solved if we analyze and compare the statistics to carve out data that might help us rearrange our expenditures. The main motto is to clarify how negligent we are when it comes to an essential thing called healthcare.

Myths and Barriers

The misconceptions and myths around public health include socioeconomic forces contributing to a significant social divide between the rich and the poor. Institutionalized, individual, and internalized inequities affect the poor [23]. They are frequently isolated and disadvantaged to the fullest extent possible. Because they do not have enough money, this intergenerational cycle of poverty continues for an extended period, with everyone experiencing increasing morbidity and less access to healthcare [24]. Gender inequality, a lack of education, hazardous and unpleasant employment, violence, and abuse directed at people of other castes or social backgrounds are all the barriers a person faces. Primary and preventive care reduces future health costs [25], but people believe prevention costs them more than different health expenditures [26]. For example, if the obesity prevention program reduces the cost of obesity-related diseases and a person gains some years because of being healthy now, the conditions he is suffering now have no relation to obesity, but that he is suffering them only because of his life expectancy being now more will offset the intention of public health investment [27].

The political mind of decision-makers is the most typical and important impediment. They believe the benefits will come much later when they may or may not be in charge (their political party might or might not be in power). So, why should they invest right now? The popularity will be gained by others and hence the lack of funding [26,28]. The identifiable victim effect is another hindrance. It is the urge to protect an individual first rather than fund an intervention that does not address the current ill health but focuses on long-term intervention for far greater people [29]. Myths such as public health is the same as clinical health, that people's health can be better in general if we build large hospitals, and that doctors alone can improve public health are just myths and must be avoided. Tools for determining priorities are essential for assisting public health decision-making processes [30].

Although the Indian public health system is lacking, specific schemes which proved their use fruitful are worth mentioning and must be recognized, giving away examples of how mixed efforts and a good solution paves the way to something prosperous and healthy. These are Reproductive, Maternal, Newborn, Child and Adolescent Health (RMNCH+A), Rashtriya Bal Swasthya Karyakram (RBSK), The Rashtriya Kishor Swasthya Karyakram, Revised National Tuberculosis Control Programme, National AIDS Control Organisation, Mission Indradhanush to improve coverage of immunization in the country, the Pradhan Mantri Swasthya Suraksha Yojana (PMSSY), Integrated Child Development Service, National Tobacco Control Programme, Rashtriya Swasthya Bima Yojana, and Pulse Polio [22]

Regarding buying power parity, India spends $215 per person, far less than other developing middle-income countries such as China, Brazil, or South Africa. Even though we have universal health coverage, it is not detailed. For example, Mexico's Seguro popular program offers low-cost solutions. It helps in less stress, a lighter psychological load, and a contented patient. In India, most avoid unnecessary treatment since out-of-pocket expenses and other hospital fees can result in severe financial difficulty [31]. Technological advancements like vital registration systems and the capacity to count births and deaths must be applied everywhere, and WHO should establish a health metric network to assist countries [17].

The Indian government has universal health coverage under sustainable development goals. It has created the Ayushman Bharat Yojana, transforming sub-centres and primary health centres into Health and Wellness Centers, but all of this is contingent on the availability of health workers, such as public health specialists. Public health specialists should be present to fight for the rights of public health investments, given the inequitable allocation [16,32-35]. By bolstering epidemiological and laboratory capabilities, the ever-present hazards of Ebola, severe acute respiratory syndrome (SARS), influenza, and dengue outbreaks can be decreased. Epidemiology training, such as that provided by the Indian epidemic intelligence service, demonstrates how early we may notice anomalous activity like epidemics. Because Indians are intelligent and have a wealth of technical knowledge, we should strive for a world-class laboratory service [16,33].

Limitations

There are a few inaccuracies in the article. When looking for resource material, the absence of well-written articles proved to be a huge setback. Although this page highlights the primary hurdles, specific solutions for specific diseases are lacking.

## Conclusions

When we methodically prepare for everyone's health, we will be able to attain it. Policymakers should make an effort to comprehend how public health is used and promote transparency. It is all right to build tertiary care facilities and purchase utilities, but not at the price of public health infrastructure. The government should give increased disease surveillance and comprehensive research the required attention to promote a healthy lifestyle. As a result, advocates for monetary investments and policymaking should receive the most support possible so that they make the right decision. Sensitization must be done because it is necessary for vibrant health, and health is achieved when people are free of disease burdens.

Government health facilities are inadequate, and a lack of staff leads to increased out-of-pocket expenditures at private hospitals. Insufficient staff indicates low quality and a lack of knowledge about equal resources for the poor and vulnerable; this makes them more vulnerable. The government should assume responsibility for assisting the poorest and most marginalized citizens. They should be able to provide incentives to doctors so that they can go to rural or tribal areas and help people. Finally, public health should not be a one-person operation. It needs to build a coalition or come under a single tent with all of the associated departments, such as community medicine, social workers, health officials, researchers, statisticians, and everyone working to make study findings and evidence generation easier. To summarize, there may be hundreds of problems and several risk factors and risks. Still, a robust, active public health system will be capable of navigating the waters if we address and identify the flaws, accept them, and try to improve them. Sustainable development goals may be impossible to attain without a solid public health system, so focusing on public health and its related needs is both necessary and ethical.
